# Repurposing AI for protein interactions and dynamics: opportunities, limitations, and lessons

**DOI:** 10.3389/fbinf.2026.1749317

**Published:** 2026-03-11

**Authors:** E. Sila Ozdemir, Hyunbum Jang, Ruth Nussinov, Attila Gursoy, Ozlem Keskin

**Affiliations:** 1 Independent Researcher, Seattle, WA, United States; 2 Computational Structural Biology Section, Frederick National Laboratory for Cancer Research in the Cancer Innovation Laboratory, National Cancer Institute, Frederick, MD, United States; 3 Department of Human Molecular Genetics and Biochemistry, Sackler School of Medicine, Tel Aviv University, Tel Aviv, Israel; 4 Department of Computer Engineering, Koc University, Istanbul, Türkiye; 5 Department of Chemical and Biological Engineering, Koc University, Istanbul, Türkiye

**Keywords:** artificial intelligence, computational approach, drug discovery, protein dynamics, protein interactions

## Abstract

Understanding protein interactions and dynamics of biological systems is central in drug discovery. Advances in artificial intelligence (AI) have expanded the scope of predictive learning for complex biological systems. Repurposing current gold-standard AI algorithms for structural and biological applications illustrates how flexible and powerful these approaches can be. In this mini-review, we examine how AI models are repurposed across domains and analyze how inductive biases, learning objectives, and representation choices inherited from their original applications shape performance in protein interaction and dynamics tasks. We discuss where AI approaches succeed, where they systematically fail, and how their behavior differs from physics-based modeling. We further highlight unresolved biological challenges, data and benchmarking limitations, and emerging opportunities for hybrid AI-physics workflows that balance efficiency with physical realism. By framing recent developments through a cross-domain adaptation framework, this review aims to provide practical guidance for selecting, evaluating, and integrating AI models in protein interaction and dynamics studies, and to support more reliable and biologically meaningful applications of AI in computational protein science.

## 1 Introduction

The past decade has seen remarkable progress in computer-aided protein science, driven not only by advances in traditional modeling approaches but also by creative integration of artificial intelligence (AI). Methods originally designed for language, image, and graph data are now being repurposed to address problems in structural biology, where they are reshaping how researchers approach protein interactions and dynamics. Here, we define repurposing as the transfer of models across domains with fundamentally different data structures, inductive assumptions, and objectives. Vision models assume spatial continuity and locality, language models (LMs) assume discrete sequences with long-range contextual dependencies, and graph learning methods assume relational structure governed by message passing. When these models are applied to proteins, they are not merely reused but are often implicitly adapted to accommodate biophysical constraints, evolutionary signals, and thermodynamic considerations. These adaptations shape both model performance and failure modes.

Prediction of protein-protein interactions (PPIs) and protein-drug interactions (PDIs) is central to understanding biomolecular mechanisms and has particularly benefited from repurposed AI models (Vakser, 2023). Diffusion-based generative frameworks, originally developed for image and audio generation, have been adapted to treat docking as a sampling problem rather than a single-pose prediction task, improving accuracy and diversity in predicted binding poses (Zhu et al., 2024). Other methods combined these diffusion frameworks with physics-informed constraints to produce chemically realistic docking predictions (Cao et al., 2025). Transformers, initially developed for natural language processing (NLP) to capture long-range dependencies in text, have been applied to protein sequences to generate embeddings that encode biochemical and structural features, i.e., multiple sequence alignment (MSA) relevant to interaction prediction (Lupo et al., 2022; Elnaggar et al., 2022). Graph neural networks (GNNs), first designed for general relational and social network data, now effectively model molecular and protein graphs to predict binding affinities, interaction interfaces, and docking poses (Zhang et al., 2022). GNNs have been adapted for docking by redesigning graph construction and feature encoding (Zhang et al., 2023). In parallel, AlphaFold (AF), although originally developed for protein structure prediction, has been rapidly integrated into biomolecular interaction prediction, docking, and virtual screening pipelines, providing high-quality structural scaffolds that inform interaction modeling (Jumper et al., 2021; Abramson et al., 2024).

**FIGURE 1:**
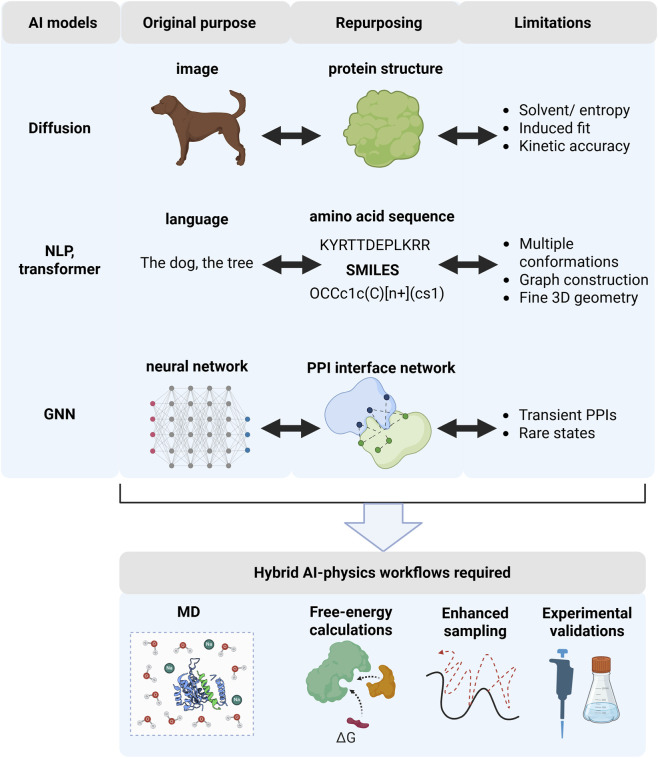
Cross-domain repurposing of AI models in protein science. Models inherit inductive biases from their original domains (left), enabling specific protein interaction and dynamics tasks (middle) but also introducing systematic failure modes after transfer (right). These limitations motivate hybrid AI-physics workflows (bottom) in which AI-generated hypotheses are refined using physics-based simulations and experimental validation. Created in BioRender.

Studying protein dynamics remains challenging due to the high dimensionality of conformational space and the cost of sampling rare or long-timescale events. Predicting PDI kinetics further compounds these challenges, as it requires accurately modeling binding and unbinding rates across multiple time scales while accounting for solvent effects, conformational flexibility, and energy landscape complexity (Ojha et al., 2024). AI approaches have enhanced molecular dynamics (MD) simulations, conformational sampling, and energy landscape modeling. Diffusion-based generative models have been adapted to learn coarse-grained force fields and accelerate exploration of conformational space (Arts et al., 2023). More broadly, AI-based language models are now being applied to accelerate sampling, extract collective variables, and reveal hidden conformational states that are often inaccessible to classical MD simulations (Khokhar et al., 2025; Do et al., 2023). These advances extend to the study of long-timescale motions, surface dynamics, and allosteric mechanisms, illustrating how coupling AI with MD can provide more accurate and scalable insights into protein flexibility and regulation. Figure 1 illustrates AI-based approaches adapted for protein science, highlighting their limitations and plausible hybrid AI-physics workflows required to address those limitations.

Taken together, these developments illustrate how AI can be applied cross-domain to advance computational biology. However, rather than summarizing AI applications in protein science by method, this mini-review advances a unifying, repurposing-centric framework that examines how AI methods originally developed for non-biological domains are transferred to protein interactions and dynamics, and how their inherited inductive biases shape model behavior, limitations, and conceptual trade-offs. In this mini-review, we focus on four key factors that determine successful repurposing. First, transferable inductive biases, such as equivariance in diffusion models, attention mechanisms in transformers, or locality in GNNs, determine which physical or biological properties are naturally captured. Second, learning objectives, including denoising, masked-token prediction, or contrastive learning, influence whether models recover structural ensembles, interaction interfaces, or dynamic transitions. Third, representation choices, such as sequences, surfaces, residue graphs, or atomic coordinates, constrain what information is accessible to the model. Finally, domain mismatch arises when assumptions from the source domain fail to hold in protein systems, for example, in modeling solvent effects, induced fit, or rare conformational states. This framework provides a critical discussion of why certain approaches succeed, where they fail, and how insights from their original domains continue to shape progress in protein interaction and dynamics modeling. Table 1 summarizes core inductive biases inherited from the original AI domains, shaping both the capabilities and limitations of repurposed models in protein science.

**TABLE 1:** Conceptual mapping of repurposed AI models from original domains to protein science applications.

Original domain	Inductive bias	Assumed data structure	Protein science task enabled	Common failure modes after repurposing
Image and audio generation	• Equivariance • Iterative denoising	• Continuous spatial grids • Manifolds	• Docking • Ensemble generation • Dynamics	• Solvent/entropy neglect • Kinetic pathway gaps • Training data bias
NLP	• Sequence modeling • Self-attention	• Discrete token • Long-range dependencies	• Interaction prediction • Functional annotation • Low-homology	• Weak energetics • Cannot resolve conformations • Evolutionary bias
Graph and network learning	• Relational message passing • Locality	Nodes, edges, neighborhood graphs	• Interface residue prediction • Affinity estimation • Allostery	• Graph construction sensitivity • Geometry loss • Limited transferability
Multimodal language and vision models	• Cross-attention • Modality alignment	Heterogeneous aligned embeddings	• DTI prediction • Multimodal interaction modeling	• Dataset bias • Poor chemotype generalization • Mechanism gaps

## 2 Cross-domain AI in protein interactions

AI techniques originally built for tasks in vision, imaging, language, or general graph data are now being repurposed to model PPIs and PDIs with increasing success. In the following sections, we apply this framework to major classes of repurposed AI models, emphasizing how domain-specific adaptations reflect underlying inductive biases.

Diffusion-based generative models exemplify repurposing through their strong inductive bias toward equivariant, continuous data generation. These models assume that complex structures can be recovered through iterative denoising on smooth manifolds. When transferred to protein science, this assumption aligns naturally with the geometric nature of molecular conformations, allowing docking and conformational sampling to be reframed as generative processes rather than deterministic optimization problems. In this setting, diffusion models progressively refine random poses of ligands or protein complexes into physically plausible binding conformations. For example, DiffBindFR frames docking as a generative problem over ligand translation, rotation, torsions, and even protein side-chain flexibility, enabling recovery of native-like poses across apo and predicted structures (Zhu et al., 2024). Also recently, AlphaFold 3 (AF3) has extended diffusion-based architectures beyond sequence modeling to predict protein-ligand and protein-nucleic acid complexes, directly supporting drug-target interaction (DTI) research by providing high-confidence three-dimensional (3D) interaction models (Abramson et al., 2024). However, solvent effects, entropy, and long-timescale kinetics are only implicitly represented. Interpreting diffusion models in protein science therefore requires recognizing both the power and the constraints imposed by their cross-domain origins.

Transformer architectures and protein language models (PLMs) represent a second major axis of repurposing, transferring inductive biases from natural language processing to protein science. When applied to proteins, this bias enables models to capture evolutionary, functional, and interaction-relevant signals from amino acid sequences alone, even in low-homology or sparse-structure regimes. PLMs such as ProteinBERT learn embeddings from sequence data that capture long-range evolutionary and functional dependencies relevant to interaction prediction (Brandes et al., 2022). Building on this foundation, Evolutionary Scale Modeling 3 (ESM3) scales training to millions of sequences, capturing evolutionary and functional relationships across diverse protein families, which enhances our ability to infer homologous interactions and generalize across different protein contexts (Hayes et al., 2025).

Building on these sequence-based representations, transformer architectures have been adapted for DTI prediction through cross-attention between protein and ligand embeddings. CAT-DTI employs cross-attention and domain adaptation to align drug and protein embeddings, improving prediction accuracy in out-of-distribution settings (Zeng et al., 2024). TransDTI leverages transformer-based language models to classify types of DTIs, showing gains on benchmark datasets (Kalakoti et al., 2022). However, the abstraction of proteins as sequences introduces inherent limitations: physical energetics, conformational heterogeneity, and solvent effects are not explicitly modeled, and multiple structural or dynamic states compatible with the same sequence cannot be distinguished without additional constraints. As a result, language model-based approaches excel at identifying interaction propensity but often require integration with structure- or physics-based methods for mechanistic fidelity. GNNs are repurposed into protein science through their core assumption that system behavior emerges from relational structure, an inductive bias originally developed for social, citation, and knowledge graphs. This relational perspective is particularly well suited for modeling PPIs, binding sites, and allosteric communication, where function arises from coordinated interactions across structured neighborhoods. Pancino et al. showcase these features by applying GNNs directly to residue-contact graphs, propagating information across local neighborhoods to pinpoint likely interaction surfaces (Pancino et al., 2024). They further demonstrate that node-level GNN classification can capture the signals necessary to distinguish interface from non-interface residues across diverse protein scenarios. On the other hand, ProInterVal validates protein-protein interfaces by distinguishing biologically relevant contacts from crystal artifacts. It also employs a GNN that learns interface representations through message passing between residues, capturing both local geometry and global topology with higher accuracy than previous interface assessment methods (Ovek et al., 2024). Beyond interface identification, GNNs have been successfully adapted for binding affinity prediction, as illustrated by ProAffinity-GNN, which models energetic contributions through message passing across curated interface graphs (20). Despite these successes, repurposing GNNs introduces sensitivity to representation choices, including graph construction rules, edge definitions, and resolution of geometric features. Inadequate encoding of 3D geometry or long-range correlations can limit generalization, particularly for flexible or transient interactions. Thus, GNN performance is highly sensitive to how biological structure is abstracted.

In sum, these examples illustrate a broader theme: diffusion models generate physically plausible poses, transformers capture long-range sequence and structural context, and GNNs map relational structure at the residue and atomic level into interaction-relevant signals, enhanced by innovations like edge-conditioned convolutions and attention mechanisms, making them powerful tools for identifying and characterizing protein-protein interfaces. Table 2 summarizes cross-domain AI approaches in protein interactions.

**TABLE 2:** Cross-domain AI approaches in protein interactions and dynamics application.

		Protein interactions application		Protein dynamics application	
AI methods	Original purpose	Tool/Model	Function	Tool/Model	Function
Diffusion	Image and audio generation	DiffBindFR (Zhu et al., 2024)	Modeling ligand and side-chain flexibility	DiffMD (Wu and Li, 2023)	Generating molecular trajectories
		SurfDock (Cao et al., 2025)	Leveraging surface geometry	ExEnDiff (Liu et al., 2025)	Producing ensembles
				Arts et al. (Arts et al., 2023)	Learning coarse-grained force fields
		AF3 (Abramson et al., 2024)	Predicting protein-ligand and nucleic acid complexes	BioEmu (Lewis et al., 2025)	Emulating equilibrium ensembles faster
NLP	Understand human language	ProteinBERT (Brandes et al., 2022)	Capturing evolutionary and structural context	DeepAllo (Khokhar et al., 2025)	Predicting allosteric sites
		ESM3 (15) (Hayes et al., 2025)	Integrating sequence and functional features		
Transformers	Sequence modeling for NLP	CAT-DTI (Zeng et al., 2024)	Linking drug and protein features	ProtTrans (Elnaggar et al., 2022)	Generating protein embeddings with structural signals
		TransDTI (Kalakoti et al., 2022)	Classifying drug-target interactions		
GNN	Graph-structured data representation	Pancino et al. (Pancino et al., 2024)	Predicting interface residues	ProAffinity-GNN (Zhou et al., 2024)	Predicting PPI binding affinity from interface graphs
		ProInterVal (Ovek et al., 2024)	Distinguishing biologically relevant contacts from crystal artifacts		

## 3 Cross-domain AI in protein dynamics

Repurposing AI models for protein dynamics extends beyond static structure prediction and exposes deeper mismatches between original domain assumptions and biological reality. Models adapted from vision and language domains typically assume equilibrium distributions or context-invariant representations, whereas protein dynamics involve rare events, non-equilibrium transitions, and history-dependent behavior. When applied to MD, diffusion models, transformers, and graph-based approaches inherit strengths in sampling and representation learning but often lack explicit mechanisms to enforce thermodynamic consistency or kinetic realism. Framing these approaches as repurposed rather than replacement methods clarifies why hybrid strategies that integrate physics-based simulations remain essential for capturing biologically meaningful dynamics. Diffusion models have proven especially effective for MD. DiffMD replaces traditional force-field integration with a score-based diffusion process, learning to directly generate molecular trajectories by iteratively denoising conformations and incorporating velocity information through an equivariant geometric transformer (Wu and Li, 2023). This eliminates reliance on potential energy or force inputs while maintaining accuracy across benchmark MD datasets, enabling accelerated simulation of folding and conformational dynamics. By iteratively “denoising” conformational noise, these models can sample structural transitions at scales unreachable by classical MD. ExEnDiff extends this idea by integrating experimental restraints into the diffusion process, generating conformational ensembles that not only reflect Boltzmann distributions but also capture mutation-induced changes and experimental observables (Liu et al., 2025). BioEmu integrates AF-derived representations with diffusion modeling to generate equilibrium ensembles orders of magnitude faster than classical MD while capturing key conformational changes (Lewis et al., 2025).

PLMs and transformers provide an additional layer by encoding information about protein flexibility and evolutionary constraints directly from sequences. Building on the insight that amino acid sequences can be treated as structured text, ProtTrans shows that embeddings learned from billions of proteins carry rich biophysical features that map onto stability and structural motifs (Elnaggar et al., 2022). Such representations have since been used to predict dynamic phenomena, such as allosteric sites and allosteric effects in DeepAllo (Khokhar et al., 2025), thereby linking static sequence information to conformational adaptability.

Finally, ProAffinity-GNN applies GNN message passing over protein interface graphs to predict protein-protein binding affinities, demonstrating accurate and generalizable performance across diverse complexes (Zhou et al., 2024). More broadly, GNN approaches excel at tracing how local perturbations, such as mutations or binding events, propagate through dynamic interaction networks, making them valuable for studying allosteric communication and flexible interfaces.

These cross-domain AI approaches are redefining how protein dynamics can be studied. Diffusion models enable efficient exploration of conformational ensembles, PLMs encode constraints embedded in sequence evolution, transformers provide a lens into context-dependent energetics, and GNNs map relational dependencies across flexible structures. By integrating these perspectives, the field is moving toward a richer, multi-scale understanding of protein dynamics that bridges static structures and dynamic function. Table 2 summarizes some cross-domain AI approaches in protein dynamics.

## 4 Comparative analysis of repurposed AI paradigms: strengths, limitations, and failures

While diffusion models, PLMs, transformers, and GNNs have all demonstrated strong performance in studying protein interaction and dynamics, their effectiveness is highly context dependent and closely tied to the inductive biases inherited from their original domains. Explicit comparison across paradigms clarifies when specific approaches are most appropriate and where their limitations necessitate complementary or hybrid strategies.

Diffusion-based generative models differ fundamentally from traditional docking and enhanced-sampling MD approaches in both objective and interpretability. Physics-based docking and MD explicitly model intermolecular forces and thermodynamic landscapes, enabling mechanistic insight and kinetic analysis but at high computational cost and with sensitivity to force-field accuracy. Diffusion models instead prioritize efficient exploration of structural space by learning data-driven generative priors, making them well suited for rapid pose generation and large-scale screening. However, because solvent effects, entropy, and long-timescale kinetics are only implicitly represented, diffusion models can produce structurally plausible poses that nevertheless lack thermodynamic or kinetic realism, particularly for rare binding events and induced-fit processes. For example, diffusion-based docking models may recover near-native binding poses with high confidence while failing to predict ligand residence time or capture slow binding and unbinding events that depend on solvent-mediated and entropic effects (Zhu et al., 2024; Cao et al., 2025; Ojha et al., 2024). In practice, these methods are best viewed as complementary, with diffusion models providing efficient initial sampling and physics-based simulations refining energetics and dynamics.

PLMs offer advantages over structure-based interaction prediction in low-homology or sparse-structure regimes, where reliable 3D models may be unavailable. By leveraging evolutionary and contextual information encoded in sequences, PLMs can infer interaction propensity and functional relevance across diverse protein families. However, this abstraction comes at the cost of physical specificity: sequence-based models cannot distinguish among multiple structural or dynamic realizations compatible with the same sequence, nor can they directly model induced fit or binding energetics. As a result, high confidence in sequence-based predictions does not necessarily imply mechanistic correctness at the structural or energetic level (Lupo et al., 2022; Elnaggar et al., 2022; Brandes et al., 2022). For this reason, PLMs are particularly effective for prioritization and hypothesis generation, while structure-based approaches remain essential for mechanistic interpretation and quantitative prediction.

GNNs occupy an intermediate position between sequence-based and geometry-explicit models. By representing proteins as relational graphs, GNNs effectively capture interface organization, residue-level communication, and allosteric pathways. Their flexibility, however, introduces sensitivity to graph construction choices, including node definitions, edge criteria, and geometric resolution as discussed above. In practice, different graph construction rules for the same protein complex can produce different interface or affinity predictions, even though the underlying biology is unchanged (Pancino et al., 2024; Ovek et al., 2024; Zhou et al., 2024). Different graph construction rules can lead to materially different predictions for the same protein system, highlighting the dependence of results on representation rather than biology alone. In contrast, equivariant geometric networks explicitly encode rotational and translational symmetries, preserving 3D structure more faithfully at the cost of increased computational complexity and dependence on high-quality structural inputs. The choice between these approaches therefore depends on whether relational abstraction or geometric precision is more critical for the task at hand.

Across paradigms, a recurring failure mode of repurposed AI models is overreliance on training distributions dominated by stable, well-characterized complexes. This limits generalization to transient interactions, intrinsically disordered regions, and non-equilibrium processes. Recognizing these shared limitations underscores the importance of hybrid AI-physics workflows and motivates the integration of experimental constraints and enhanced sampling into future modeling strategies.

## 5 Discussion

Recently, AI methods adapted from other domains are reshaping how we model interactions, conformational landscapes and dynamic processes. These approaches do not simply replicate existing techniques; instead, they bring fundamentally new ways of learning from data, capturing long-range dependencies, and generalizing across complex structural states. Despite these advances, challenges remain. Diffusion models and PLMs often rely on simplified or static representations that can give an impression of predictive accuracy while masking important physical and kinetic limitations. The performance of repurposed AI models is strongly constrained by the availability and diversity of well-curated structural datasets. Recent efforts, compiling thousands of experimentally determined 3D PPI interface structures, mapping all experimental structures to the reference human proteome, and curating dataset of drug-like molecules bound to interfaces, have greatly expanded the available training landscape (Abali et al., 2024; Cankara et al., 2024; Kosoglu et al., 2023).

Despite rapid progress, several biologically important classes of protein interactions and dynamic processes remain poorly captured by current AI approaches. Transient and weak PPIs, interactions involving intrinsically disordered regions, and complexes stabilized primarily by solvent-mediated or entropic effects are consistently underrepresented in training data and challenging for data-driven models to infer. Similarly, rare but functionally critical conformational transitions, such as ligand-induced allosteric switching or slow binding and unbinding kinetics, are difficult for repurposed AI models to capture because these events violate equilibrium and smooth-manifold assumptions inherited from their original domains. Recognizing these failure modes is essential to prevent misinterpretation of AI outputs as mechanistic insight. In these settings, physics-based simulations often remain more reliable, as they explicitly model energy landscapes and time evolution. These examples illustrate that AI models often perform best when predicting stable, well-represented complexes and struggle in precisely the solvent-driven, transient, and kinetically complex regimes that define many biologically relevant interactions (Ojha et al., 2024; Abali et al., 2024; Cankara et al., 2024; Kosoglu et al., 2023).

A major bottleneck in the effective deployment of repurposed AI models is the lack of standardized, task-specific benchmarks that reflect biologically realistic conditions. Many commonly used datasets are biased toward stable, well-characterized complexes and high-affinity interactions, limiting model generalization to weak, transient, or context-dependent binding. Furthermore, benchmark metrics often emphasize structural similarity or classification accuracy without assessing physical plausibility, kinetic realism, or robustness under perturbation. For protein dynamics, benchmarking remains particularly underdeveloped. Few datasets provide ground-truth ensembles or kinetic observables suitable for validating AI-generated trajectories. Without consistent evaluation standards, it is difficult to compare AI models against enhanced-sampling molecular dynamics or to quantify the trade-offs between speed and physical accuracy. Addressing these gaps will require coordinated efforts to develop shared benchmarks, uncertainty-aware evaluation metrics, and datasets that span a broader range of interaction strengths and dynamic behaviors. Meaningful benchmarks for protein interaction and dynamics modeling should include weak and transient binders in addition to high-affinity complexes, as these interactions are common in biological systems but underrepresented in current datasets. For dynamic modeling tasks, benchmarks should ideally provide ensemble-level ground truth rather than single static structures, allowing evaluation of conformational diversity and state transitions. Incorporation of kinetic observables, such as binding and unbinding rates or residence times, would enable assessment of whether AI models capture biologically relevant timescales rather than only structural similarity. In addition, uncertainty estimates and robustness to perturbations should be considered as evaluation criteria, as high-confidence predictions may still lack physical or mechanistic validity. Without such properties, benchmarks risk rewarding models that perform well on well-behaved, static systems while failing to reflect performance in biologically realistic settings.

Rather than viewing AI and physics-based simulations as competing paradigms, the most promising advances are emerging from hybrid strategies that leverage the strengths of both. AI models excel at rapid exploration of high-dimensional conformational space and at learning priors from large datasets, while physics-based methods provide mechanistic grounding, thermodynamic consistency, and interpretability. Hybrid workflows are particularly well suited for problems such as docking with induced fit, where AI-generated poses can initialize enhanced-sampling MD; for conformational ensemble generation, where diffusion models propose candidate states refined by free-energy calculations; and for allosteric modeling, where graph-based or LM-derived signals guide targeted simulations. These approaches reduce computational cost while preserving physical rigor, making them attractive for large-scale screening and hypothesis generation followed by focused mechanistic validation.

To support responsible and effective use of repurposed AI models in protein interaction and dynamics studies, we propose the following practical considerations: First, model selection should be guided by the biological question rather than performance on generic benchmarks. Sequence-based models are most appropriate for prioritization and inference under limited structural information, whereas structure- or geometry-aware models are better suited for mechanistic interpretation. Second, representation choices should be made explicit and justified, as graph construction, resolution, and feature selection strongly influence model behavior. Sensitivity analyses that test robustness to representation changes can help identify spurious dependencies. Third, AI predictions should be evaluated not only against static structural metrics but also for physical plausibility, stability under perturbation, and consistency with experimental or simulation-based observables when available. Finally, AI outputs should be treated as probabilistic hypotheses rather than definitive predictions, particularly in regimes involving rare events, non-equilibrium dynamics, or sparse data. Integrating AI predictions with physics-based refinement and experimental validation remains essential for drawing reliable biological conclusions.

From a practical perspective, the choice of AI model should be guided by the biological question rather than benchmark performance alone. When structural information is limited, sequence-based models are most effective for prioritization and functional inference, but not for mechanistic interpretation. For docking and conformational sampling, diffusion-based approaches offer efficient pose generation that should be followed by physics-based refinement for energetic validation. GNN-based methods are particularly useful for interface and allosteric analysis when graph construction faithfully reflects biological geometry, and sensitivity analyses are recommended to ensure robustness. Benchmark accuracy should not be interpreted as biological validity; AI outputs should be evaluated for physical plausibility and stability under perturbation. In practice, AI predictions are most valuable when treated as hypotheses that guide targeted simulations and experiments within hybrid AI-physics workflows.
